# Crown Ether-Immobilized Cellulose Acetate Membranes for the Retention of Gd (III)

**DOI:** 10.3390/polym13223978

**Published:** 2021-11-17

**Authors:** Oana Steluta Serbanescu, Andreea Madalina Pandele, Madalina Oprea, Augustin Semenescu, Vijay Kumar Thakur, Stefan Ioan Voicu

**Affiliations:** 1 Department of Analytical Chemistry and Environmental Engineering, Faculty of Applied Chemistry and Materials Science, University Politehnica of Bucharest, Gheorghe Polizu 1-7, 011061 Bucharest, Romania; oana.sserbanescu@yahoo.com (O.S.S.); pandele.m.a@gmail.com (A.M.P.); madalinna.calarasu@gmail.com (M.O.); 2 Advanced Polymers Materials Group, University Politehnica of Bucharest, Gheorghe Polizu 1-7, 011061 Bucharest, Romania; 3 Faculty of Materials Science, University Politehnica of Bucharest, Splaiul Independentei 313, 060042 Bucharest, Romania; augustin.semenescu@upb.ro; 4 Academy of Romanian Scientists, Splaiul Independentei 54, 030167 Bucharest, Romania; 5 Biorefining and Advanced Materials Research Center, SRUC, Edinburgh EH9 3JG, UK; 6 Department of Mechanical Engineering, School of Engineering, Shiv Nadar University, Uttar Pradesh 201314, India; 7 School of Engineering, University of Petroleum & Energy Studies (UPES), Uttarakhand, Dehradun 248007, India

**Keywords:** cellulose acetate, crown ether, gadolinium retention

## Abstract

This study presents a new, revolutionary, and easy method of separating Gd (III). For this purpose, a cellulose acetate membrane surface was modified in three steps, as follows: firstly, with aminopropyl triethoxysylene; then with glutaraldehyde; and at the end, by immobilization of crown ethers. The obtained membranes were characterized by Fourier transform infrared spectroscopy (FT-IR) and X-ray photoelectron spectroscopy (XPS), through which the synthesis of membranes with Gd (III) separation properties is demonstrated. In addition, for the Gd (III) separating process, a gadolinium nitrate solution, with applications of moderator poison in nuclear reactors, was used. The membranes retention performance has been demonstrated by inductively coupled plasma mass spectrometry (ICP-MS), showing a separation efficiency of up to 91%, compared with the initial feed solution.

## 1. Introduction

Although researchers have a high interest in Gd (III), its applications and methods for its retention [1,2,3,4,5,6,7], the literature is inferior in methods for retaining this element and in methods for controlling this element’s concentration in solution. This is also the case since the main applications of gadolinium-based salts are strictly regulated. Gadolinium-based salts have two main applications. They are used as contrast agents for clinical nuclear magnetic resonance (NMR) [8,9] and as moderator poison in nuclear reactors for fission reaction control [10], but small amounts of complexed Gd (III)-based solutions may remain in the brain, causing toxic effects [11,12], or need to be removed entirely from the nuclear reactor moderator system. The use of Gd (III) in the nuclear field is limited to a single practical application, such as in nuclear reactors reactivity control or shutdown, by changing gadolinium nitrate concentrations in the moderator system. To adjust Gd (III) concentration in the moderator water, which is used to support nuclear chain reactions, a single solution is applied: ion exchange resins, based on polystyrene [13], with a retention efficiency ranging from 150 to 178 mg Gd (III)/g resin. A greater retention efficiency can be obtained using a mixed bed resin column (M.B.), consisting of a strong acid cationic resin (SAC) and a strong base anionic resin (SBA) [14], for a retention efficiency of 250 mg Gd (III)/g of resin. New functionalized mesoporous silica systems [15,16] or magnetic nanoparticles composites systems [17] have been tested, but ion exchange remains the technical solution with the highest retention efficiency. Recently, a membrane system for Gd (III) retention has been developed, based on cellulose acetate functionalized with calmagite, a membrane with two remarkable adjacent properties—Gd (III) retention by calmagite complexing reaction and membrane surface color change, indicating the retention process [18].

Now, membranes are unique materials, due to their unique property, unpossessed by any others—selectivity [18,19]. A membrane is a functional material that essentially works as a barrier to chemical species, but is permeable to others, in a multi-component system [18,20]. Other than the separative ones, many niche fields have been developed lately with polymeric membranes in the center. Applications, such as tissue engineering [21,22,23,24] or membrane reactors for oxidative photodegradation of organic compounds, have found increasingly pronounced applicability, detrimental to classical membrane processes [25,26]. Methods of modeling the separative properties of membranes include the use of surfactants added to the polymer solution [27], the synthesis of composite membranes in which the filler takes an active role in the separation process [28,29,30,31], and the synthesis of functionalized membrane materials [32,33,34]. 

The present study was performed to separate Gd (III) from water, using cellulose acetate membranes with immobilized crown ethers. Gadolinium nitrate is used to control nuclear reactions, which, in the last years, has successfully replaced boric anhydride for this [18,35,36,37]. Given that nuclear solutions are of high purity, the possibility of having and separating other interferences from the system was eliminated from the beginning [18,38]. It is essential to separate gadolinium from the moderator water to control nuclear reactivity in the design manual specification limits.

## 2. Materials and Methods

The membranes were obtained from a cellulose acetate (Sigma Aldrich, St. Louis, MO, USA, analytical reagent) solution in N, N′ dimethylformamide (Sigma Aldrich, St. Louis, MO, USA, analytical reagent) (12% wt.), by phase-inversion method, and were precipitated in a mixture of water and ethanol at 1:1 ratio (vol.). To modify the membrane surface, we used our previous study about cellulose acetate membranes surface modification, in which the following steps were presented: hydrolysis of acetyl groups to increase the number of hydroxyl groups at the membrane surface; immobilization of aminopropyl triethoxysilane (APTES) and the reaction with glutaraldehyde (GA) at APTES amino groups; followed by crown ethers immobilization. The crown ethers immobilization was performed at –OH groups, similar to the resveratrol immobilization previously reported [39].

**Figure 1:**
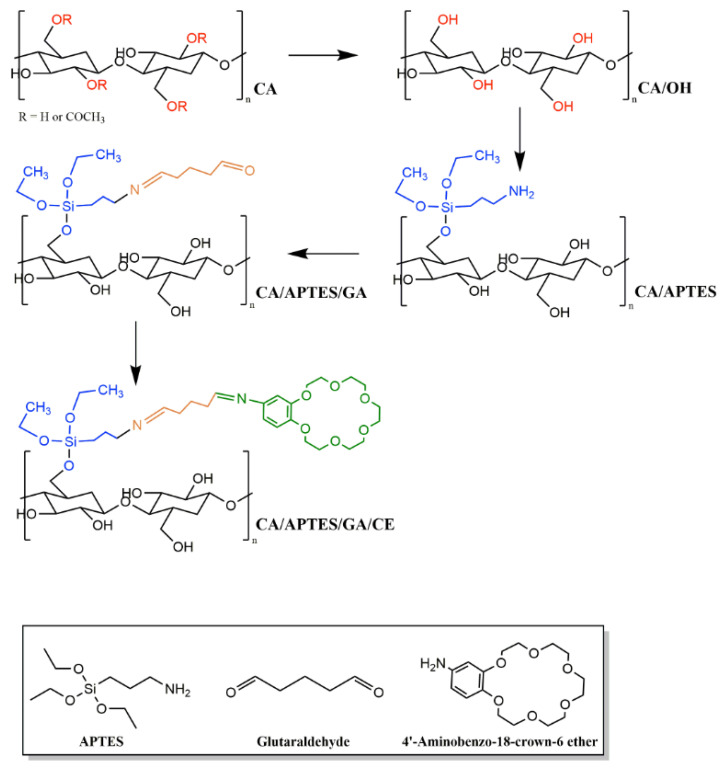
Reaction scheme for immobilization of crown ether on cellulose acetate membranes.

For modifying cellulose acetate membranes surface with APTES (Sigma Aldrich, St. Louis, MO, USA, analytical reagent) and GA (Merck, Kenilworth, NJ, USA, analytical reagent), our study follows the sericine and resveratrol immobilization process previously reported in the literature [40]. A sodium hydroxide solution is used for partial membrane hydrolysis, the APTES immobilization reaction being catalyzed by a weak base (a 0.1 N sodium hydroxide solution). After the reaction had taken place, the membranes were washed with deionized water to remove the remanent APTES. Glutaraldehyde was used to bind APTES to crown ethers. After functionalization, the membranes are washed, and cold stored, using ultrapure water to avoid microorganisms’ formation and proliferation on the membrane surface. The reaction scheme is shown in Figure 1.

For Fourier transform infrared spectroscopy analyses (FT-IR), we used Bruker Vertex 70 (Bruker, Billerica, MA, USA) equipment, with a diamond ATR device in the range of 600–4000 cm^−1^. After 32 successive measurements, an average spectra result was reported, eliminating noise, atmospheric CO_2_, and atmospheric moisture.

The cellulose acetate and modified cellulose acetate membranes surface structure analyses were performed by X-ray photoelectron spectroscopy (XPS), using a K-Alpha instrument from Thermo Scientific (Thermo Fischer, Waltham, MA, USA), with a monochrome source of Al Kα (1486.6 eV), at a pressure of 2 × 10^−9^ mbar. The absolute calibration of the binding energy scale was performed using Au 4f_7/2_ (reference binding energy 83.96 eV), Ag 3d_5/2_ (reference binding energy 368.21 eV), and Cu 2p_3/2_ (reference binding energy 932.62 eV). The pass energy for the scattering spectra was 200 eV [18,41,42,43]. The fastest scanning degree was 1 eV, and the lowest for high-resolution spectra was 0.1 eV.

The flow rates were determined using a Sartorius module with 47 mm membrane discs and 250 mL distilled water.

The water permeance (***Wf***) in L m^−2^ h^−1^, was calculated using the following formula (Equation (1)):

(1) $$Wf=\frac{V}{A\times t}$$

where ***Wf***—water flow; ***V***—feed solution volume (L); ***A***—membrane area (m^2^); and ***t***—time (h).

Gadolinium retention efficiency was determined using a feed gadolinium nitrate solution in ultrapure water (1 g/L). The analysis was performed by ICP-MS, using Bruker equipment (Bruker, Billerica, MA, USA). Gadolinium retention efficiency was calculated using the following formula:

(2) $$R=100-\frac{C_{F}}{C_{P}}\times 100\;$$

where ***R***—Gd (III) retention (%); ***C_F_*** and ***C_p_***—Gd (III) concentrations in the feed and permeate solutions, respectively (g·L^−^^1^).

## 3. Results

Between the polymers used to obtain polymeric membranes, cellulose and its derivatives occupy a special place, with the first membranes ever obtained having been obtained from nitrocellulose. Cellulose derivatives have the great advantage of being soluble in a wide range of polymer solvents, this fact offering versatility when obtaining membranes by phase inversion or solvent evaporation. However, the fields of applicability of these membranes are limited due to the low chemical and mechanical resistance, especially the danger of hydrolysis in too acidic or too basic environments, with the breaking of the polymer chains and the decrease of the mechanical and hydrodynamic resistance of the membrane.

The cellulose derivative membranes’ chemical and mechanical resistance and selectivity can be improved by functionalization and derivatization reactions. The use of APTES proved to be an ideal molecule for immobilizing other species on cellulose derivative membranes, also having the advantage of reticulation of the membrane surface with a role in its stabilization [44]. APTES was used for the immobilization of magnetic particles [45], the compatibilization of nanocellulose with polyethersulfone [46] or polyvinyl alcohol [47], the hydrophobization of cellulose-based fabrics [48,49], or the reactive retention of dyes for wastewater purification [50,51,52,53].

For the application presented in this study, cellulose acetate, despite its chemical or mechanical properties not necessarily being remarkable, can be an ideal candidate because, from a chemical point of view, the cooling solution of a nuclear reactor is as clean as possible in terms of the content of compounds that could vary in acidity or basicity, being a solution that contains the majority of Gd (III), respectively, among a series of other cations.

**Figure 2:**
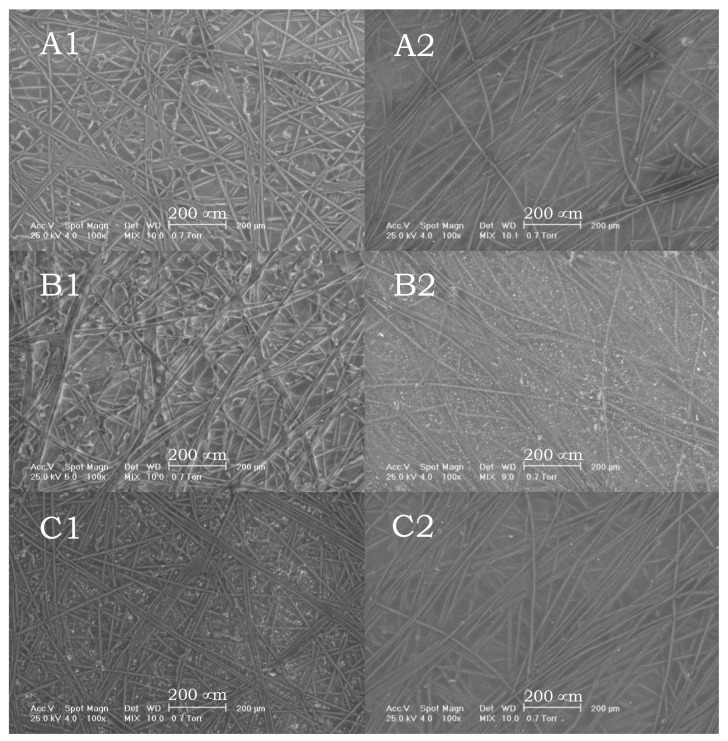
SEM images of active surface for CA neat membrane (**A1**), CA/APTES/CE membrane (**B1**), and CA/APTES/CE membrane after Gd (III) filtration (**C1**) and porous surface for CA neat membrane (**A2**), CA/APTES/CE membrane (**B2**), and CA/APTES/CE membrane after Gd (III) filtration (**C2**).

SEM images (Figure 2) revealed very interesting modifications between neat CA membrane, functionalized membrane with crown ethers, and functionalized membrane after Gd (III) retention. The active layer of the neat CA membrane is characterized by a fibrillar structure with intercalated fibers in a random arrangement. The same morphology can also be observed on the porous surface of the membrane, but with higher spaces between fibers (due to the asymmetric structure of the membranes). After the immobilization of crown ethers, a crosslinking effect can be observed on both surfaces due to the functionalization with glutaraldehyde, which partially reacts with both groups, an observation also supported by the TGA analysis. After Gd (III) filtration, the crosslinking effect is more pronounced, this being explained by the complexation reaction for retention, which can imply more than one crown ether molecule. Due to the large atomic volume of Gd (III), two or more crown ether molecules can consequently complex the cation, this fact leading to a concentration of polymeric fibers and a more compact structure on both surfaces of the membrane. The observation is also supported by the retention of Gd (III), which dramatically increases at the end of retention, an increase explained by this compactness of the surface. 

**Figure 3:**
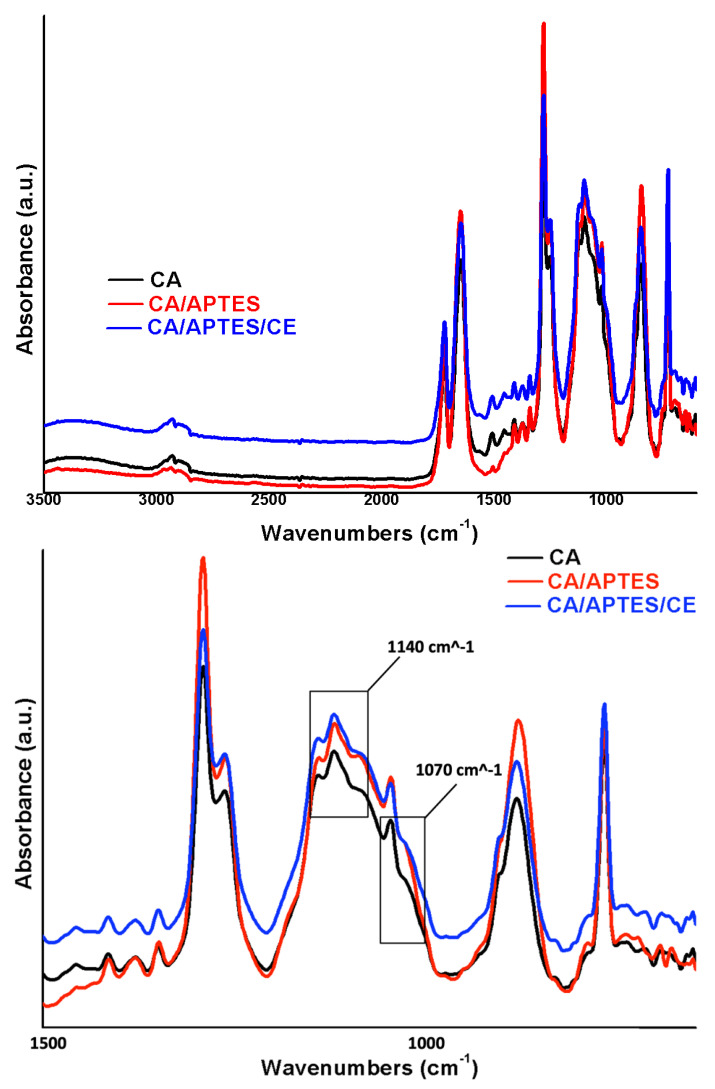
Fourier transform infrared spectroscopy spectra (FT-IR) of synthesized membranes CA, CA/APTES, and CA/APTES/CE, respectively.

The FT-IR spectra (Figure 3) proves the functionalization of the synthesized membrane. After partial hydrolysis of cellulose acetate membranes, to increase the number of hydroxyl groups at the surface of the membrane, the specific peak for C=O decreases in intensity because the number of acetyl groups was reduced during the hydrolysis process. Furthermore, the functionalization with APTES causes a displacement of all peaks because of the Si atoms presence. Immobilization of crown ethers caused a change in the intensity of all bands (due to the functional group’s modifications as against the total surface of the membrane), as well as their slight displacement (for the same reasons). A detailed discussion of the modification steps was provided in the previous work, which describes the steps of the modification of the membrane with APTES and GA, the novelty here is represented by the complexant molecule for Gd (III)—crown ethers [18]. Because in all synthesis strategy stages are implied the same functional groups, it is difficult to make a complete discussion for functionalization considering only the FT-IR investigation. At a closer look to the spectra can be easily observed that changes in intensity and small shifts appear for all bands that characterized synthesized membrane materials.

The reaction scheme presented is an ideal one, being almost impossible to evaluate how every molecule of APTES will react with the polymer [18,53].

The immobilization of crown ethers on the CA membrane can be observed in the IR spectrum of the CA/APTES/CE where, in addition to the corresponding cellulose acetate peaks, the presence of bands at 1070 (stretching vibration of C-O) and 1140 cm^−1^ (stretching vibration of C-O-C) of the CE structure can be observed with small shifts and changes intensity. 

**Figure 4:**
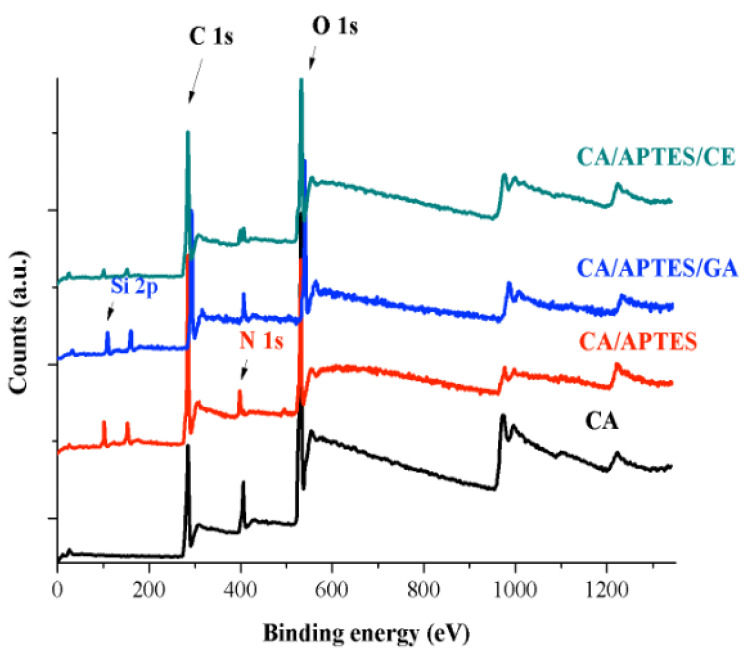
XPS analyses of membranes: analysis of CA, CA/APTES, CA/APTES/GA, and CA/APTES/CE, with marked peaks of interest for C 1s, O 1s, Si 2p and N 1s.

XPS survey spectra are presented in Figure 4, and the C 1s, O 1s, N 1s and Si 2p atomic percentages for each sample are provided in Table 1. XPS analysis showed significant changes in the surface atomic composition between neat cellulose acetate membrane and membranes after the next modification steps. The main atoms identified on the surface of obtained membrane materials are C 1s (at 287 eV), O 1s (531 eV), N 1s (at 400 eV), and Si 2p (at 102 eV). XPS CA spectrum presents three peaks for C 1s (45.16%), O 1s (44.8%), and N1s (6.86%). The remanent solvent (N, N’—dimethylformamide) can explain the presence of nitrogen from membrane synthesis. After APTES immobilization, a percent of 8.64% Si 2p was found at the surface, while the other elements percentage are modified according to partial hydrolysis of CA, followed by the reaction with APTES. It must be noticed that at XPS survey analysis, the modification can be evaluated considering not only the chemical modification steps at the membrane surface but also the steric and geometric arrangements of molecules in their integrity (atoms and bond angles, etc.). After reaction with glutaraldehyde, the C 1s percentage increased by 70.31% due to the crosslinking effect on the surface, which led to a high concentration of C atoms at the surface of the membrane. For the last functionalization step—the immobilization of crown ethers—in comparison with CA/APTES/GA, the percent of O 1s increased from 20.27% to 33.21% due to the presence of the six oxygen atoms in every crown ether molecule and also by the orientation of ethylene groups at the membrane surface. At the same time, the nitrogen is no longer observed, and the silicon percent decreased to 2.83%. This can be explained by the aromatic ring in the crown ether molecule, which led to a shield effect on the membrane surface for the atomic structures found under the layer of crown ethers [11]. 

**Figure 5:**
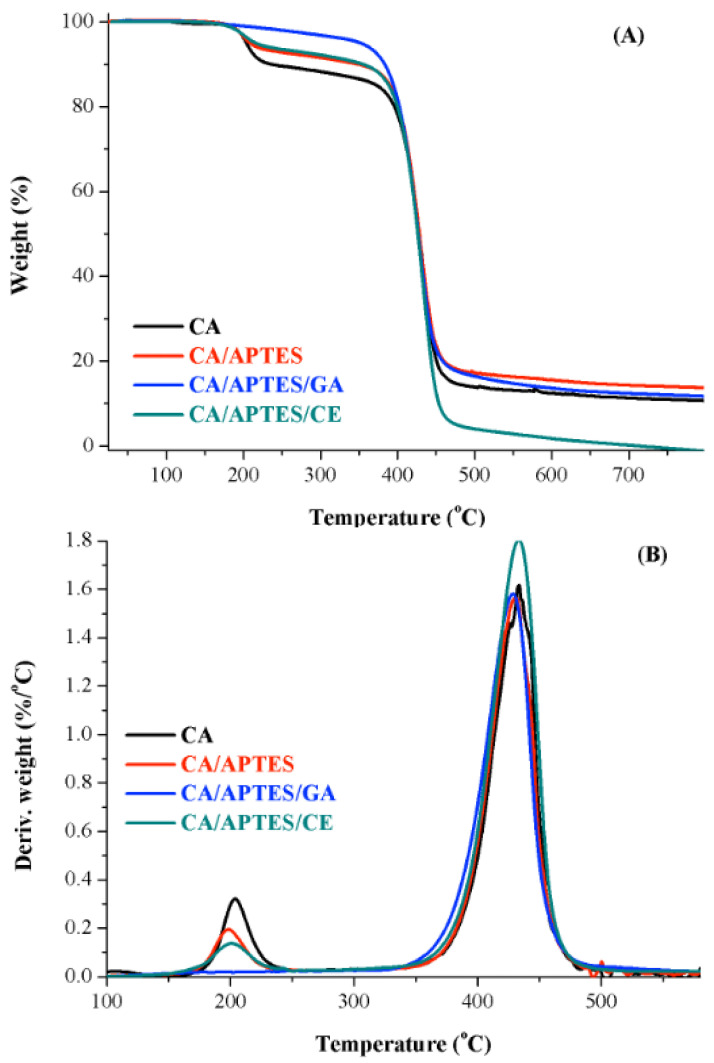
TGA (**A**) differential thermal analysis and (DTA) (**B**) spectra of CA, CA/APTES, CA/APTES/GA, and CA/APTES/CE membranes.

The thermostability of the materials was studied through the TGA and DTG curves (Figure 5) and the data are presented in Table 2. According to the figure, most of the samples illustrate two degradation stages, as follows: the first one is due to the evaporation of the solvent and the second one is due to the decomposition of the polymeric chain. An exception is made by CA/APTES/GA samples that show a single degradation step. This is due to the GA molecules immobilized on the membrane surface, which has the same reactivity at both functional groups, leading to a crosslinking effect at the membrane surface. The best thermostability was recorded in the case of the CA/APTES/GA membrane, and this is due to the cross-linking effect obtained on the surface of the membrane following the immobilization of GA. A drop in thermostability was observed for CA/APTES/CE membrane. To immobilize CE molecules, one functional group is re-transformed into -CHO, followed by CE derivatization. Similar results were obtained in previous studies, where we managed to immobilize tetracycline [32] or resveratrol molecules on the CA membrane’s surface [39]. Regarding the DTG curves, no significant changes could be observed, which means that the synthesized membranes were stable during the modification process.

**Figure 6:**
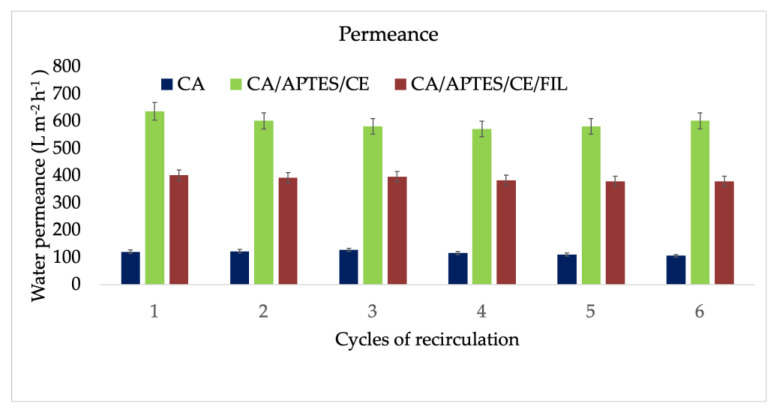
Permeance after 5 recirculation cycles. The errors were calculated using 5 membranes to evaluate the homogeneity of the materials obtained.

In addition, the water permeance through synthesized membranes was studied (Figure 6). For the neat CA membrane, the initial flow was around 160 L m^−2^ h^−1^. The small decrease for each cycle can be explained by the hydrodynamic stabilization of the membrane determined by the compactness of the polymer chains inside the porous layer under the pressure of water. After functionalization with crown ether, the flow rate increased to about 680 L m^−2^ h^−1^ due to the hydrophilic character of etheric groups. Simultaneously, the membranes show variable stability from cycle to cycle due to the same hydrodynamic stability for the porous layer of the membrane. More stable behavior was observed for the membranes that already retained Gd (III), which presented a permeance of around 400 L m^−2^ h^−1^. The highest stability can be explained by the membrane structure being already stabilized during retention at the pass of water through membrane pores. The decrease in permeance was determined by the presence of Gd (III) ions with high atomic volume that acts as a foulant at the active layer surface. For every cycle the time varied, the only constant being the volume of feed solution—250 mL.

**Figure 7:**
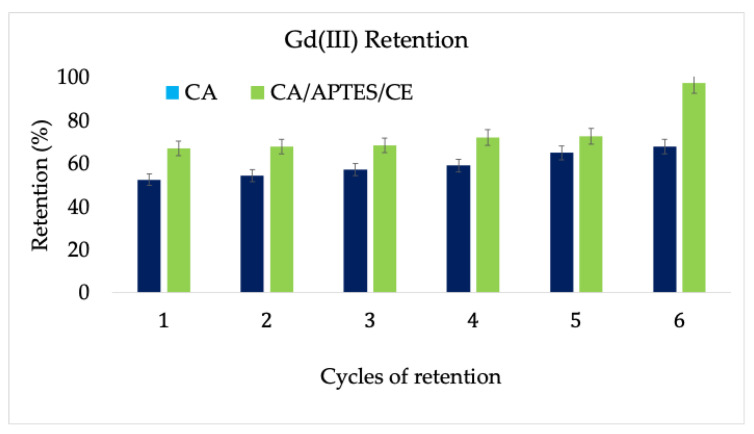
Retention of Gd (III) through the cellulose acetate control membrane and through the membrane functionalized with crown ether. The errors were calculated using 5 membranes to evaluate the homogeneity of the materials obtained.

The total retention (Figure 7), assessed by ICP-MS, was 91% for the functionalized membrane, compared with 64% for the standard cellulose acetate membrane. It can be observed that, in the first 5 cycles, for the CA/APTES/CE membrane, the increase in retention is not very significant, starting from 68% at the first cycle, increasing to 78% at the 5th; however, in the 6th cycle, the retention dramatically increased to 91%. This difference can be explained by the complexing reaction of the immobilized crown ether to the surface of the membrane. This can occur between one, two, or even three crown ether molecules that could complex Gd (III), which would lead to a crosslinking of the membrane surface. The same effect was observed in the previous reported retention of Gd (III) with calmagite, but in that case, a maximum of two calmagite molecules were able to complex Gd (III) ions, which explains the lower maximum retention at 86% [18]. Practically, at the 6th cycle, a maximum fouling or a critical one at the surface of the functionalized membrane is achieved, which conducts to this value of retention. 

## 4. Discussion

The membranes consecrated for ion exchange are usually obtained from polysulfone by introducing anionic or cationic functional groups in the polymeric chain. The advantage of this polymer is primarily related to the mechanical and thermal properties of polysulfone, being one of the most resistant polymers. On the other hand, it is well known that polysulfone membranes have negligible ion-exchange capacity [33]. After amination, for example, the ion-exchange capacity increases drastically, the membrane being able to retain Cr (VI) ions [54] efficiently. The sulfonation reaction is another example through which one can induce an ion-exchange character to a polysulfone membrane [55,56,57], being known to the versatility of this functional group at the complexation of metal cations. In addition, chloromethylation can provide ion exchange capacity to membranes and versatility for subsequent functionalization reactions that allow membranes the capacity for complexation processes for cations retention [58,59,60]. The nanofiltration process would represent another technological solution, but it raises some practical problems. First, the energy consumption associated with this process is very high, and the operating costs are implicitly higher. Second, the membranes used should be composite membranes with nanofillers that increase the separation efficiency (such as graphene), which would significantly improve both the costs of raw materials and the costs of synthesis of composite membranes.

The philosophy that justifies this research is not only represented by cost diminution of the technological solution for retention of gadolinium nitrate (used for the control of nuclear reactivity), but also by a transition from synthetic polymers (even if we are referring to polysulfone ion exchange resins or other technical polymer membranes) to the use of cellulose derivatives (a natural polymer, which is sustainable, from renewable resources). In the first reported literature study of a synthesized membrane for Gd (III) separation from water, which moderates a nuclear reactor (reported by the same research group), the basic idea of separation is a complexation reaction. Cellulose acetate membranes were functionalized with calmagite—a metallic color indicator with the capacity of complexing cations. The presence of indicator assured an extra property to membranes—the capacity of self-indicating the retention of Gd (III) through the change of membrane surface color. Practically, the self-indication of separation efficiency was another premiere in the field of membrane science, with no additional analysis methods being necessary. Immobilization of crown ethers on the same cellulose acetate-based membrane (keeping same steps of synthesis until immobilization of final species) leads to a superior separation yield due to the capacity of crown ethers to complex cations in a much easier manner than calmagite (explicable by the presence of the six oxygen atoms, each with free electron pairs). Compared with calmagite immobilized membranes—where one or two complexant molecules were necessary for a retention reaction—in the case of crown ethers, this mechanism can occur between one, two, or even three complexant molecules that could complex Gd (III), which would lead to a crosslinking of the membrane surface. Still, it is worth mentioning that the use of crown ethers for separations by complexing reactions presents a high lack of selectivity since the ethers will complex any cation present in the feed solution. Immobilization of crown ethers also opens the possibility for other membranes applications, especially in the biomedical field. Custom-made hemodialysis membranes for the retention of heavy metals can be obtained in the same manner, or this could be simply a way to increase the membranes’ hemocompatibility. If the membranes are saturated with Ca^2+^ ions, this could lead to an increased hemocompatibility, given that it is well known that the presence of Ca^2+^ ions can regulate the thrombocytes behavior. Moreover, the same membrane, saturated with Ca^2+^ ions, can be used in osseointegration applications, with this favoring the mineralization of natural bone and integration of an implant into the bone. 

## 5. Conclusions

This research is practically the second report in literature on a membrane that can separate Gd (III) from water, with previously reported results exclusively based on ion exchange resins. For this purpose, the surface of a cellulose acetate membrane was modified in several stages: initially with the aminopropyl triethoxysylene, followed by the reaction with glutaraldehyde, and the immobilization of crown ether. The synthesized materials were characterized by infrared spectroscopy with Fourier transformant (FT-IR) and X-ray photoelectron spectroscopy (XPS), which demonstrated the synthesis of the membrane with separation properties of Gd (III). In comparison with previously published results, reported by the same research group, the total retention capacity of the membrane was evaluated at 86% from the initial Gd (III) solution by the functionalization of the membrane with crown ether, and a 91% efficiency of separation was achieved. Immobilization of crown ethers on the same cellulose acetate-based membrane (keeping the same steps of synthesis until the immobilization of final species) leads to a superior separation yield due to the capacity of crown ethers to complex cations in a much easier manner than calmagite (explicable by the presence of the six oxygen atoms, each with two free electron pairs).

## Figures and Tables

**Table 1:** XPS atomic percentages obtained for the analyzed samples.

Sample	C 1s [%]	O 1s [%]	N 1s [%]	Si 2p [%]
CA	45.16	44.80	6.86	-
CA/APTES	64.96	20.49	5.91	8.64
CA/APTES/GA	70.31	20.27	4.44	4.98
CA/APTES/CE	63.27	33.21	-	2.83

**Table 2:** The wt (%), Td5%, and the DTG values for the CA membrane, before and after the modification process. Three different samples were analyzed for calculating the standard deviation.

Sample Name	Wt. (%)	DTG (°C)	Td_5%_ (°C)
CA	89 ± 1	433 ± 1	204 ± 3
CA/APTES	86 ± 1	431 ± 1	209 ± 3
CA/APTES/GA	88 ± 1	430 ± 1	353 ± 3
CA/APTES/CE	100 ± 1	433 ± 1	216 ± 3

## Data Availability

Not applicable.
